# Opportunities and challenges in deriving phytoplankton diversity measures from individual trait-based data obtained by scanning flow-cytometry

**DOI:** 10.3389/fmicb.2014.00324

**Published:** 2014-07-01

**Authors:** Simone Fontana, Jukka Jokela, Francesco Pomati

**Affiliations:** ^1^Department of Aquatic Ecology, Eawag, Swiss Federal Institute of Water Science and TechnologyDübendorf, Switzerland; ^2^Department of Environmental Systems Sciences, Institute of Integrative Biology (IBZ), ETH ZurichZurich, Switzerland

**Keywords:** biodiversity, environmental change, ecosystem functioning, scanning flow-cytometry, individual level data, traits, functional diversity, biodiversity indices

## Abstract

In the context of understanding and predicting the effects of human-induced environmental change (EC) on biodiversity (BD), and the consequences of BD change for ecosystem functioning (EF), microbial ecologists face the challenge of linking individual level variability in functional traits to larger-scale ecosystem processes. Since lower level BD at genetic, individual, and population levels largely determines the functionality and resilience of natural populations and communities, individual level measures promise to link EC-induced physiological, ecological, and evolutionary responses to EF. Intraspecific trait differences, while representing among the least-understood aspects of natural microbial communities, have recently become easier to measure due to new technology. For example, recent advance in scanning flow-cytometry (SCF), automation of phytoplankton sampling and integration with environmental sensors allow to measure morphological and physiological traits of individual algae with high spatial and temporal resolution. Here we present emerging features of automated SFC data from natural phytoplankton communities and the opportunities that they provide for understanding the functioning of complex aquatic microbial communities. We highlight some current limitations and future needs, particularly focusing on the large amount of individual level data that, for the purpose of understanding the EC-BD-EF link, need to be translated into meaningful BD indices. We review the available functional diversity (FD) indices that, despite having been designed for mean trait values at the species level, can be adapted to individual-based trait data and provide links to ecological theory. We conclude that, considering some computational, mathematical and ecological issues, a set of multi-dimensional indices that address richness, evenness and divergence in overall community trait space represent the most promising BD metrics to study EC-BD-EF using individual level data.

## General introduction

Microbial ecology is currently undergoing a dramatic change, with potential implications for theory and practice in general microbiology, community and ecosystem ecology. New technologies (e.g., secondary-ion mass spectrometry, flow-cytometry, next generation sequencing, and single cell genomics) are uncovering vast genetic and functional diversity (FD), and novel microbial groups and functions (Prosser et al., 2007; Rinke et al., 2013; Wessel et al., 2013). Such accumulation of data requires guidance of sound theory and the application of robust analytical tools to provide mechanistic insight and, ultimately, predictive power that is of practical and cross disciplinary value (Prosser et al., 2007). The application of theory added by an *ad-hoc* analytical testing tools is currently limited in large datasets produced by microbial ecologists.

Here we focus on the challenge of understanding and predicting the effects environmental change (EC) on biodiversity (BD), and the consequences of BD change for ecosystem functioning (EF) (Hillebrand and Matthiessen, 2009; Reiss et al., 2009; Cardinale et al., 2012). Diversity provides functionality and stability of ecosystem processes, overall determining the resilience and adaptive capacity of an ecosystem to change (Norberg et al., 2001; Loreau, 2010; Naeem et al., 2012). Comprehensive reviews in several fields of ecology and ecosystem science have highlighted a multitude of research needs in EC-BD-EF context (Naeem and Wright, 2003; Arkema et al., 2006; Suding et al., 2008; Cianciaruso et al., 2009; Hillebrand and Matthiessen, 2009; Reiss et al., 2009; Naeem et al., 2012). The pressing knowledge gaps include, among others, the need to introduce a trait-based analysis using the characteristics of individual phenotypes rather than species, to create measures of FD that relate to system processes and selection/evolution of traits as they respond to EC. Diversity of functional traits, as opposed to taxonomic diversity, appears to be a better predictor of EF across a range of communities (Suding et al., 2008; Hillebrand and Matthiessen, 2009; Reiss et al., 2009; Cardinale et al., 2012). Species-derived BD metrics may not be directly linked to EF, and the relationships between, for example, species richness and FD or EF are not trivial (Naeem and Wright, 2003; Petchey and Gaston, 2006; Cianciaruso et al., 2009; Cardinale et al., 2011).

Monitoring a large number of traits with potential functional properties in a natural microbial community is a requirement for understanding the effects of EC on the BD-EF relationship, but presents a number of great challenges. Given the fast generation time of most microorganisms and the relatively small spatial scale at which events occur, this task requires high-frequency sampling of fluctuating small-scale environments (Pomati et al., 2011; Stocker, 2012). Scanning flow-cytometry (SFC) and recent developments in its applications give promising tools for the automated counting, characterization and identification of aquatic planktonic microorganisms. Aquatic microbial communities are a classic model in EC-BD-EF research, since they control a significant part of global biogeochemistry and roughly half of the primary production on earth (Falkowski, 2012; Stocker, 2012). In this article we review the literature of FD indices and we advocate that environmental flow-cytometry offers the opportunity of pioneering the development of BD indices that describe phytoplankton FD starting from individual level trait data, and of testing of the linkages between these FD indices, EC and important community processes.

## An individual trait-based perspective to BD-EF research

Species still play a leading role in microbial ecology experiments and theory, even though a number of recent findings challenge the existence of a prokaryote species in nature (Ereshefsky, 2010; Rinke et al., 2013). A species centered view blindfolds the importance of phenotypic variance within and between populations and its importance in ecological and evolutionary response of populations and communities to EC. Traits, defined as any morphological, physiological or phenological feature measurable at the individual level (such as cell size, shape, motility, nutrient uptake requirements, type of reproduction) (Reiss et al., 2009), offer a “common currency” to expand BD-EF theory since trait-based approaches, by focusing on phenotypes, have the potential to incorporate variance within and between populations in the observed BD patterns, to capture time-dependent responses associated with EC, and link their effects on community-level and ecosystem processes (Norberg et al., 2001; Norberg, 2004; Suding et al., 2008; Hillebrand and Matthiessen, 2009).

Four components of an individual level trait-based research offer crucial links with EC and EF. First, individual trait variation accounts for intra-population variance, therefore it captures the range of available phenotypic plasticity in trait expression, i.e., it forms the needed trait-based link between individual genotypes and variability in their aggregated responses. Second, trade-offs among traits constrain how selection and evolutionary processes influence higher order effects in response to EC. A hierarchical expansion from individuals to community level in measures of continuous distributions of phenotypic values (traits) would offer the opportunity to apply concepts and theories from evolutionary ecology and genetics (e.g., Norberg et al., 2001) to understand consequences of trait evolution for EC-BD-EF research. Understanding how evolutionary responses to EC reflects on BD-EF is at the moment largely missing. In order to achieve this, it will be necessary to separate phenotypic from genetic responses, which may be accomplished in the near future by single-cell genomics (Rinke et al., 2013), by experimentally measuring heritability of observed phenotypic expression, or through experimental evolution tests. Third, metrics of population-and community-wide trait-distributions allow linking ecological processes (such as environmental filtering, stochastic population dynamics, and species interactions) to individual responses and EF (Norberg et al., 2001; Savage et al., 2007; Pomati et al., 2013). Fourth, functional trait diversity is dependent on the spatial and temporal turnover of individuals and populations within a community, and represents the range of strategies that a system can express to respond to fluctuating selection and dynamic ecological change. Dynamics of functional trait diversity therefore should relate to stability, resilience and adaptive capacity of the ecosystem (Norberg et al., 2001; Norberg, 2004; Isbell et al., 2011). Plasticity in trait variation can buffer populations from extreme temporal fluctuations in the environment and in population density providing mechanism for resilience, and accounts for niche and functional responses on community effects that do not appear significant when considering species-mean traits (Bolnick et al., 2011).

## Application of automated SFC in the field: current use and main limitations

**Figure 1:**
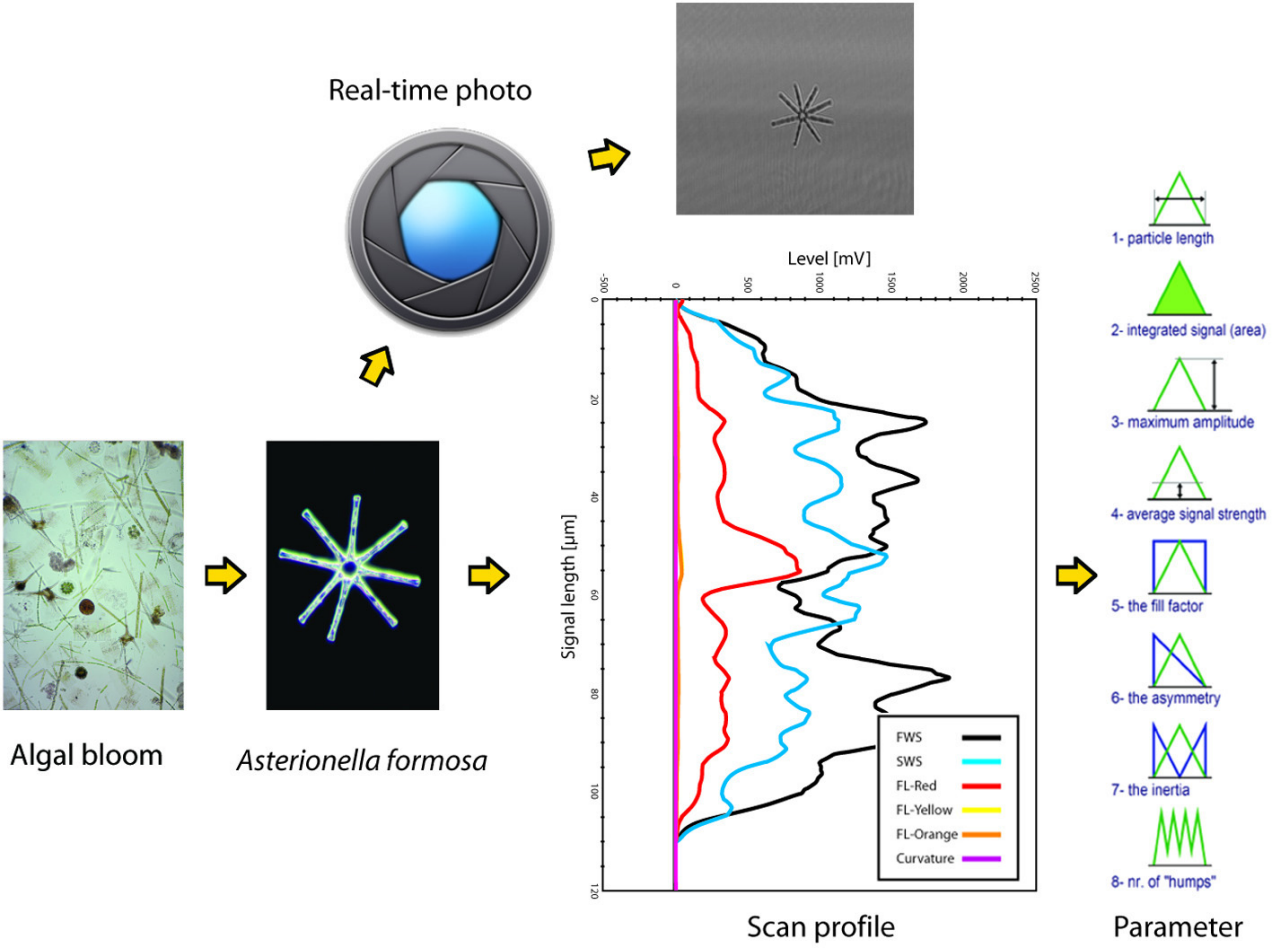
**Schematic summary of the scanning flow-cytometry approach to phytoplankton particle characterization, as operated by the Cytobuoy (Dubelaar et al., 1999; Dubelaar and Jonker, 2000)**. Particles in a water sample are separated by the instrument's injector and internal fluid systems (not depicted in the figure) and then scanned by one or more lasers. Signals from different detectors (FWS, forward scattering; SWS, sideward scattering; FL-red, chlorophyll-a fluorescence; FL-yellow and -orange, fluorescence from accessory or degraded pigment) are recorded for each particle in a time-resolved mode (scan-profile). The example shows the scan of a colony of *Asterionella formosa*, note its accurate description by the sensors. Each scattering and FL scan-profile can be studied for several parameters that describe its length, area, amplitude, symmetry etc., representing a description of the particle's shape and pigmentation. The Cytobuoy also allows to photograph scanned particles, potentially providing additional information at the morphological and taxonomical level.

SFC can measure individual microbial cells and colonies since its lasers and optical system allow sensor measurements (light scattering at different angles and fluorescence) in a time-resolved mode (scan-profiles) (Figure 1). In the flow-cell, particles are aligned along their main axis, and their identical flow-speed provide a highly accurate laser-scan (Figure 1). The parameterization of scattering and fluorescence (FL) scan-profiles results in a very fine and reproducible characterization of three-dimensional and FL particle descriptors, when particles are larger than the diameter of the laser beams (circa 5 μm). Particles smaller than the width of the laser beams cannot be properly characterized by their scan-profiles, so SFC for small particles provides information similar to conventional flow-cytometry, such as total (integrated signal) for scattering and FL allowing distinctions for example in pigment profiles, with the addition of an accurate estimation of particle length down to circa 1 μm of size.

The performance of SFC in different applications has been documented (Dubelaar et al., 1999; Maltsev, 2000; Dubelaar et al., 2004; Sosik et al., 2010). Besides laboratory use, recent developments in SFC allow also *in-situ* use: samples are taken fully automatically at any time or sequence of times set by the operator. In particular, SFC as provided by the commercially available Cytobuoy (www.cytobuoy.com) (Dubelaar et al., 1999; Dubelaar and Jonker, 2000) allows the automated sampling and analysis of microbial plankton communities in marine (Thyssen et al., 2007, 2008a,b, 2011) and freshwater (Pomati et al., 2011; Arnoldini et al., 2013) systems. Additionally, automated SFC can be integrated with multi-parametric sensors for aquatic ecosystem monitoring (e.g., measuring water chemistry, physics and algal pigments) in order to link phytoplankton BD with EF (productivity) and EC over time or over the vertical profile of a deep water body (Dubelaar and Jonker, 2000; Sosik et al., 2010; Pomati et al., 2011).

As an example of SFC data, we will focus on data collected by a Cytobuoy flow-cytometer (Figure 1). The datasets may typically comprise up to tens of thousands of individually scanned particles per aquatic sample. Scaled up to complete monitoring datasets, we can reach the level of millions of scan-profiles and associated algal descriptors. The size and complexity of such datasets leads to special challenges in handling, processing, visualization and analysis, especially considering that no standardized techniques for this purpose exist to date. Each particle can be described by 48–54 parameters (depending on the number of available lasers) based on the light scattered at two angles (forward and sideward, providing information on size and shape of the particles) and FL emitted by photosynthetic pigments (chlorophyll-a, phycocyanin, phycoerythrin, and degraded pigments) (Dubelaar et al., 2004; Malkassian et al., 2011; Pomati et al., 2011) (Figure 1). Parameters measured with Cytobuoy instruments are the amplitude, length, FL patterns and shape of these signals, and are governed by the morphology and pigmentation of particles: in the case of phytoplankton, they potentially represent physio-morphological traits such as size, pigment type, pigment concentration and pigment distribution within cells or colonies, coloniality, internal cell rugosity (cytoplasmatic structures like vesicles and membranes) (Thyssen et al., 2008b; Pomati et al., 2013). The Cytobuoy also has the capability to take photographs of particles that flow through the laser beam, potentially providing information at the morphological and taxonomical level for large nanoplankton (10–20 μm) and microplankton (20–200 μm) (Figure 1). The current low resolution of photos prevents investigation of smaller cells and fine image analysis, however particles dimensions and potentially biovolume can be estimated.

As mentioned in the previous chapter, SFC partially underestimates the diversity of pico-planktonic organisms (size range between 0.2 and 2 μm), for example small eukaryotic flagellates. We suggest that the lack of high resolution on scan-profiles for small cells is not a major limitation of SFC analysis of phytoplankton diversity. In general, the most common pico-phytoplankton are rather similar with regards to their morphology, with more important ecological differences in pigment profiles (which can be accounted for by total FL signals). We believe that there are stronger limitations in the trait-diversity analysis performed by SFC than the low resolution on morphology of pico-plankton, for example the lack in SFC data of physiological (e.g., nutrient acquisition) and life history traits (e.g., type of reproduction, resting stages) that can convey very important ecological and evolutionary information.

### Deriving biodiversity measures

**Figure 2:**
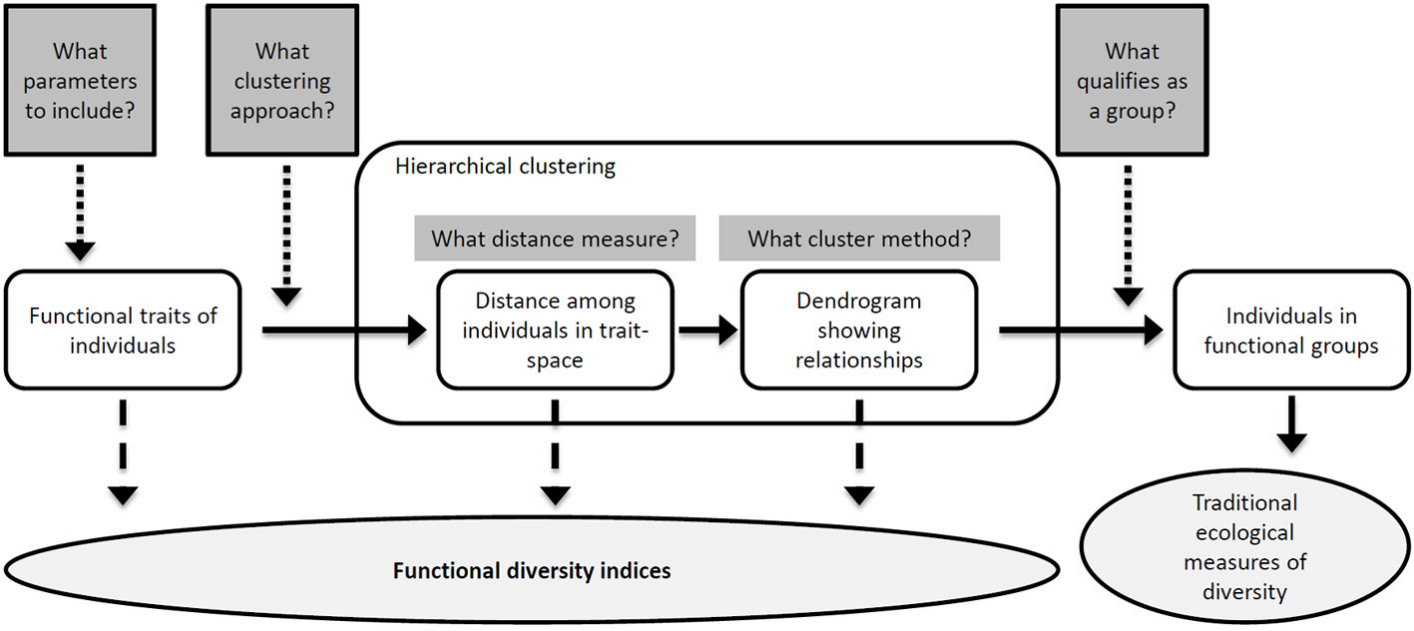
**The process of producing a functional classification (unshaded objects) of individuals in natural community samples**. At different steps of the sequential process, which contains a number of critical decisions, different measures of FD (shaded ellipse) can be estimated (see section Concluding Remarks and Outlook and Table 1). The shaded rectangular boxes represent decisions in the process of making a classification, so that the number of decisions required for each measure increases from left to right. Adapted from Petchey and Gaston (2006).

Parameters measured with Cytobuoy are partially redundant, since many morphological and FL descriptors are cross-correlated. Therefore, the choice of measured parameters represents the first critical decision step in the process of defining BD measures (Figure 2). A first assessment of traits based on correlation and ecological relevance is required in order to extract a number of robust descriptors for individual particles. Fortunately, phytoplankton is a classical model in ecology and evolution, and detailed knowledge about its most relevant ecological traits exists, which can also be relatively easily validated with microscopic analysis. In particular, cell size and shape (which influence motility and nutrient uptake through surface-to-volume ratio), and photosynthetic performance (driven by pigment type and concentration), are key phytoplankton traits, affecting growth, metabolism, access to resources, and susceptibility to grazing (Litchman and Klausmeier, 2008). Previous work has shown that a number of size- and pigment-related parameters monitored by Cytobuoy analysis are ecologically meaningful, responding to changes in nutrient levels, temperature, and abundance of zooplankton grazers (Pomati et al., 2011, 2013). Many of these focal traits, such as size and pigment content, also appeared to be under selection by environmental filters and species interactions, determining changes in population dynamics and phytoplankton community functioning (Pomati and Nizzetto, 2013; Pomati et al., 2013).

With individual-based data such as SFC's, conventional ecological measures of BD are not directly applicable. One possible approach is to define groups based on some key traits or on a set of linear combinations of all traits (for example, principal components) (Pomati et al., 2011, 2013) (Figure 2). In this context, we suggest to utilize a set of defined and well understood phytoplankton traits for studying BD changes, rather than principal components (which would be specific to the dataset), in order to allow for comparison of results across studies. A classification of phytoplankton into categories based on morphological characters has shown to offer good prospects in terms of objectivity, reproducibility, functional properties, and prediction (Kruk et al., 2010). In order to create functional categories of organisms, different approaches have been proposed for clustering SFC datasets (Caillault et al., 2009; Malkassian et al., 2011; Pomati et al., 2011, 2013). Groups obtained by statistical clustering seem to retain meaningful ecological information, since SFC-based clusters and associated traits have shown distinct diurnal and seasonal dynamics (Thyssen et al., 2008b, 2011; Pomati et al., 2011). Additionally, the total abundance within these categories can be tracked and characterized by conventional species-based metrics (such as richness, evenness and diversity indices) (Figure 2). In previous work, we have found that patterns of Cytobuoy-derived functional groups were comparable to those of identified phytoplankton taxa, both with regards to alpha- and beta-diversity measures (Pomati et al., 2013).

We also noted that the identity (and abundance) of Cytobuoy-derived functional groups did not fully reflect the identity (and abundance) of microscopically defined taxonomic groups (Pomati et al., 2011, 2013). Several species can in fact map into one functional category (in case they share similar morphology) and individuals of the same species can be assigned to different groups (single cells vs. colonies, for example), hampering our ability to fully interpret the observed ecological dynamics. This phenomenon can also be explained by the much larger volumes analyzed by traditional microscopic methods for the analysis of phytoplankton (hundreds of mL) compared to those sampled by automated SFC (few mL). The likelihood to count large particles is higher in microscopic-based methods, while small cells are more efficiently counted by flow-cytometry. To account for this different resolution of flow-cytometry across the range of phytoplankton abundances and sizes it is possible to perform two separate protocols in SFC to analyze small (and abundant) particles on one side, and large (and rare) particles on the other side. This approach has the limitation of not providing a single comprehensive description of the phytoplankton community in terms of the distribution of organisms in the multi-dimensional trait-space and their relative abundance. Such a structure carries important information about the ecological and evolutionary processes that have shaped the community, and should be the target (see below) of trait-diversity indices. The alternative strategy is to calibrate sampling protocols in order to retain only particles above the analytical scanning limit (1 μm) and sample volumes larger enough to acquire sufficient information about the characteristics of both small and large particles and cover the widest possible trait-diversity of the phytoplankton. This approach has the limitation of losing information about pico-plankton smaller than 1 μm, but retains the actual community structure in terms of relative abundance of organisms and their descriptors in the multidimensional trait-space.

Current approaches employed for BD research using SFC data have two main scientific limitations. First, they require statistical clustering or artificial neural networks to construct functional groups (Boddy et al., 2001), and these final categories may not have a general ecological interpretation. Each different method suffers from arbitrary decision steps that may critically affect the outcomes in terms of BD metrics, through identity and abundance of resulting formed groups (Figure 2) (Petchey and Gaston, 2006). Second, SFC data do not yet fully exploit the rich individual-based description of communities embedded within the datasets and described above. Individual-based SFC data are in the form of high-dimensional multivariate distributions of phytoplankton descriptors, which have variable strength as predictors of the relevant target processes. As reviewed in section An Individual Trait-Based Perspective to BD-EF Research, there is an emerging consensus that the composition and distribution of continuous measurable functional traits, i.e., FD, is a better predictor of EF, stability and adaptive capacity of the system compared to richness or evenness of groups of organisms (Norberg et al., 2001; Naeem and Wright, 2003; Norberg, 2004; Hillebrand and Matthiessen, 2009; Reiss et al., 2009). Below we review the available metrics that directly take into account the full range of multiple trait variation for quantifying FD for each given community (Mouchet et al., 2010; Schleuter et al., 2010). Most FD metrics and indices have rarely or never been applied to individual level data, used in empirical studies, or tested for their temporal and spatial relationships with important community processes. We argue that SFC data represent a unique opportunity to experiment and develop individual-level FD metrics.

## Trait-based biodiversity indices and metrics

Classical BD measures (species richness and diversity indices) treat individuals within a species as identical (Magurran and McGill, 2011), i.e., ignoring their phenotypic variance. For individual-level data, we need richness and diversity metrics that include and account for intraspecific differences. Although in the last years several FD indices and metrics have been proposed (Table 1), and some of them can take into account intraspecific trait differences, none of these metrics has been specifically designed for application to large and complex (e.g., multiple traits) individual-based datasets that may or may not include taxonomic information. Table 1 summarizes available FD metrics and the main reasons for excluding them to in the application to individual-based trait data. In the following sections we analyze and discuss the requirements that metrics have to fulfill to be further considered in an individual-based context.

**Table 1:**
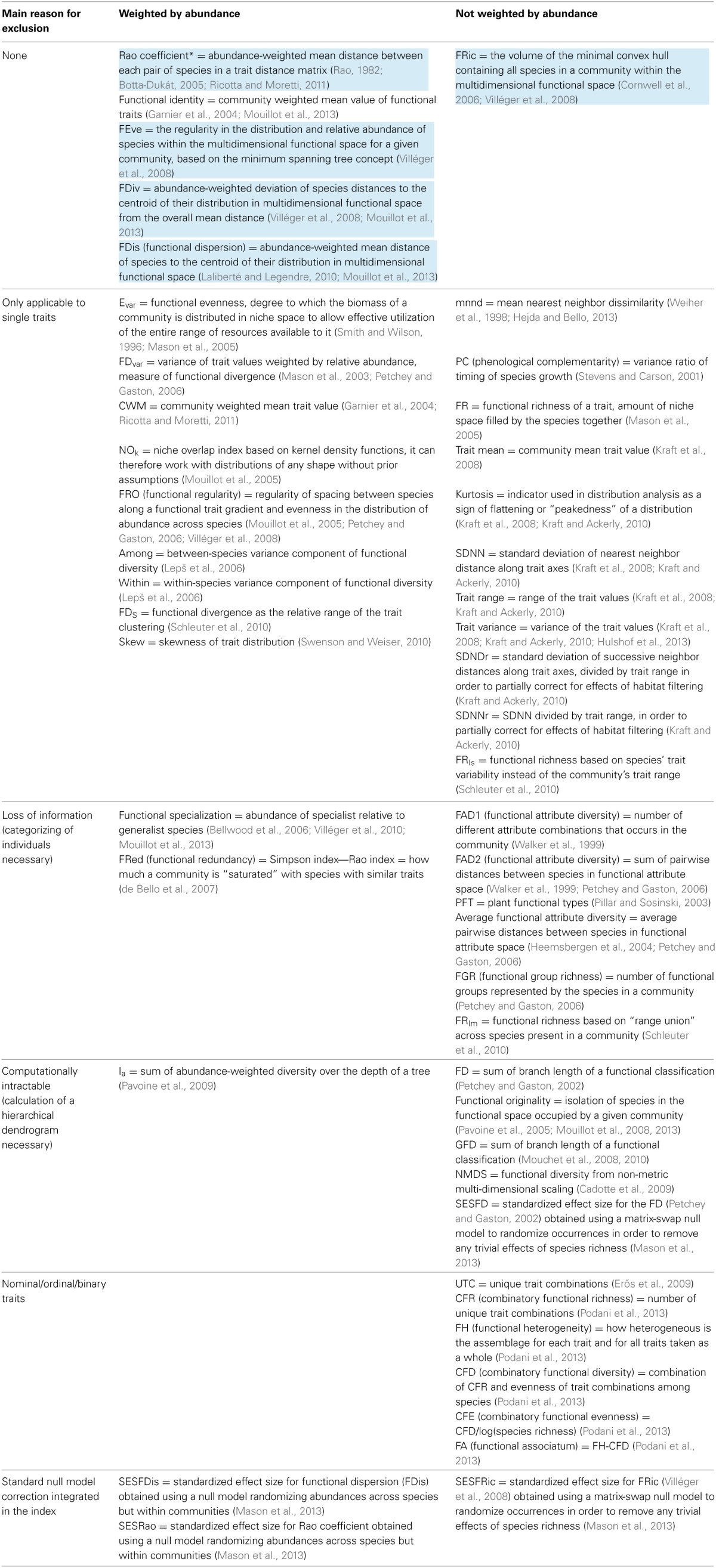
**List of published functional metrics and associated references**.

*Functional metrics are subdivided based on the eventual reasons for excluding their application to individual-based trait data and further classified according to their potential to be weighted by individual taxa abundance. The most promising functional metrics for application to individual-based SFC data are highlighted in blue boxes*.

^*^Mean distance/dissimilarity among species (MPD) represents the abundance-weighted mean phylogenetic or trait dissimilarity among all possible pairs of species (Warwick and Clarke, 1995; Pavoine and Bonsall, 2011; de Bello et al., 2012) and is therefore analogous to the Rao coefficient. However, it excludes same-species pairs and this is not meaningful in an individual-based context, where each individual is unique and species are not defined.

One classification criterion of published metrics is their potential to be weighted according to abundance of individual taxa (Table 1). In the context of species level data, abundance weighting allow considering not only the position of species in the multi-dimensional trait space, but also their relative frequency. In the case of traits measured at the individual level, abundance weighting is not necessary, since every point in multi-dimensional space represents one single organism. Abundance is therefore already embedded within the multivariate description of the community. Abundance weighting does not however affect the validity of a metric for its application to individual-based trait data. In indices that can take into account relative abundance, this can be set to value 1 and the metric applied to individual level data.

### Why should multiple traits be preferred

Published functional metrics are based either on single or multiple traits. One-dimensional indices account for selection on single traits by EC, and have been successfully utilized to test hypotheses on the processes that determine diversity, identity, and abundance of co-occurring species. This approach allows a direct link to classic ecological theory: responses in the distribution of single traits at the community level (convergence on similar values, reduction of trait range, clustered patterns, evenly spaced traits) can help inferring mechanisms such as environmental filtering, competitive exclusion of some species, grazing/predation/parasitism by specialists or generalists, competition for resources (Macarthur and Levins, 1967; Chesson and Kuang, 2008; Kraft et al., 2008; Cornwell and Ackerly, 2009; Ingram and Shurin, 2009; Kraft and Ackerly, 2010; Paine et al., 2011; Pomati et al., 2013). For example, species co-occurrence patterns can be partially explained by habitat filtering, niche partitioning and stabilizing processes (Kraft and Ackerly, 2010), and even different trophic groups seem to be reciprocally linked by the response of single morphological traits to ecosystem management (Moretti et al., 2013). A single trait approach, however, does not afford an comprehensive view of species differences in potentially important functional traits, which may affect functioning directly or indirectly (Suding et al., 2008). Additionally, when studying niche complementarity, results based on a single trait are biased toward finding functional redundancy among species, because functional niches that are distinct in a multi-dimensional functional space could appear as overlapping when only a trait (dimension) is considered (Rosenfeld, 2002; Mouchet et al., 2010).

Metrics based on multiple traits allow a more comprehensive view of differences among taxa, and potentially among individuals. A multi-dimensional trait approach is common in ecological morphometrics, classical ecology, evolutionary genetics and evolutionary ecology, where it is used to illustrate differences between biological entities (e.g., morphs, niches, genotypes, and life-history strategies). Multiple trait metrics should theoretically offer a better link to ecosystem function, because the community contribution to ecosystem processes results from the combination of all traits of species or individuals (Suding et al., 2008; Reiss et al., 2009). The use of complementary multiple trait metrics has recently been proposed as a powerful framework for detecting responses to disturbance, and to relate them to ecological theory (Mouillot et al., 2013). For instance, multi-dimensional FD provided support for the physiological tolerance hypothesis in plant communities (Currie et al., 2004), which represents a specific case of assembly through environmental filtering (Cornwell and Ackerly, 2009), suggesting that increased species richness in favorable climatic conditions is a consequence of availability of a wider range of functional traits (Spasojevic et al., 2014). We argue that FD indices based on multiple traits are better suited for linking BD metrics to EC and EF using individual-based data. This is especially true if we consider that a multi-dimensional approach still allows to separately consider single trait responses, while one-dimensional indices lack the evaluation of individual differences in a multi-dimensional trait space.

### Further criteria for application to individual-based SFC data

Some mathematical aspects in the calculation of indices can be simplified when dealing with traits measured at the individual level using SFC. For example, we suggest not to consider those indices that integrate mathematical methods for separating intra- from interspecific trait differences (Schleuter et al., 2010), since individual-based data already combine both hierarchical levels. In addition, we suggest to exclude indices that need and assume trait frequency distributions or other data necessarily aggregated at the species level, because in SFC data every single individual represent a unique combination of traits and the process of categorizing would cause unavoidable loss of potentially important information (Table 1). Finally, we consider only the indices that have been designed to deal with traits that are continuous or expressed as proportions (i.e., not nominal, ordinal or binary traits), as for SFC measurements such as size and FL typically belong to this kind (Table 1).

Individual-based SFC datasets, especially if collected automatically with high spatial or temporal resolution, are generally extremely large. One possible way to solve this problem would be develop efficient subsampling algorithms, able to reduce the amount of data points maintaining the topology of data in a multi-dimensional space and the relative abundance of organisms. Suitable FD indices should however be computationally efficient. It could be convenient, for example, to avoid the calculation of distances and functional dendrograms among individuals (Petchey and Gaston, 2002). These require computation of metrics of similarity/dissimilarly among all data points and construction of topologies that become intractable for normal computers given the very large matrices (Table 1). The limitation of such approach becomes even more relevant when it's necessary to compare different samples (communities) across space and time, since for this purpose it is generally necessary to build an overall distance matrix and functional dendrogram merging all samples in a single dataset. Considering that it would also be desirable to minimize the number of arbitrary and critical decision steps in calculating FD indices (Figure 2), we argue that the best FD measures should also be robust toward possible methodological issues.

Since we seek to understand links between EC, BD dynamics and EF, we do not necessarily have to consider indices that incorporate a standard null model correction (Table 1) (Mason et al., 2013). Indeed, randomization of matrices may not be the best null approach in many cases since neutral (stochastic) processes may not generate strictly random patterns (Bell, 2005). Suitable indices to study FD and EF should rather allow their application to individual-based neutral models that simulate stochastic processes, such as dispersal of individuals, demography (birth, death, and reproduction) and ecological drift (Allouche and Kadmon, 2009; Jabot, 2010; Vanpeteghem and Haegeman, 2010).

### Functional richness, evenness, and divergence

Some BD indices represent a combination of different components of FD. FD_var_ (Mason et al., 2003), Rao coefficient (Rao, 1982; Botta-Dukát, 2005), and FDis (Laliberté and Legendre, 2010) combine measures of functional richness and divergence (Mason et al., 2013). In order to adequately describe and understand individual-level FD, however, each one of the ideally suitable multi-trait indices should measure one of the three different components of FD: richness, evenness, and divergence (Mason et al., 2005). In the most ideal case, these components are independent to each other (orthogonal). Additional components of FD have been suggested (Podani et al., 2013), they however do not apply to potential individual level trait data. FD components such as combinatory functional diversity (CFD) or functional heterogeneity (FH) only concern trait combinations in the context of nominal traits (i.e., non-quantitative traits coded into categories by discrete numbers), while we focus on continuous high-resolution data (Table 1). Another metric, though not effectively being a FD index, could be useful in characterizing communities in a multi-dimensional trait space. The functional identity (multi-trait analogous of community weighted mean CWM; Table 1) represents the centroid (coordinates) of a trait distribution in multi-dimensional functional space (Garnier et al., 2004; Spasojevic and Suding, 2012; Mouillot et al., 2013). With centroid coordinates information it is possible to detect a uniform translation of all data points in space, case in which FD indices of richness, evenness, and divergence are not supposed to change. However we argue that, at least in the context of multi-dimensional individual trait data, most of the changes in community functional structure would be reflected in functional richness, evenness and divergence.

Functional richness is the total amount of space occupied by all taxa of a community in the multi-dimensional trait space. An ideal richness index should be able to take into account and exclude gaps in trait distribution. In other words, trait richness approximates as accurately as possible the multi-dimensional space effectively occupied by the community, rather than a simple trait range. Nevertheless, very different estimates of functional richness have been proposed, such as the volume of the minimal convex hull containing all taxa within the multi-dimensional space (Cornwell et al., 2006; Villéger et al., 2008), the total branch length of a dendrogram calculated according to distances between taxa in the trait space (Petchey and Gaston, 2002) or simply the number of functional groups represented by taxa in a community (Petchey and Gaston, 2006).

Functional evenness is a measure of how evenly taxa are distributed within the trait space. While different one-dimensional indices have been proposed for even spacing of traits, e.g., E_var_ (Smith and Wilson, 1996; Mason et al., 2005), FRO (functional regularity) (Mouillot et al., 2005) and SDNN/SDNNr/SDNDr (based on the standard deviation of nearest or successive neighbor distance) (Kraft and Ackerly, 2010), only one multi-dimensional index of functional evenness based on quantitative data exist (Table 1). FEve measures the regularity in the distribution of taxa within the multi-dimensional functional space, calculating a network that links all the points with the minimum sum of branch lengths (minimum spanning tree) (Villéger et al., 2008).

Functional divergence measures how spread-out taxa are in the multi-dimensional trait space. For its estimation several approaches are possible. FDiv quantifies how single taxa distances to the centroid of a trait distribution in the multi-dimensional space deviate from the overall mean distance (Villéger et al., 2008). The Rao coefficient is the total distance between each pair of taxa in a trait distance matrix (Rao, 1982; Botta-Dukát, 2005; Ricotta and Moretti, 2011), while FDis (functional dispersion) measures the mean distance of taxa to the centroid of a trait distribution in a multi-dimensional space (Laliberté and Legendre, 2010; Mouillot et al., 2013).

### A possible way forward

We suggest that to study EC-BD-EF relationships in general, it is necessary to select a set of FD indices that are able to account for physiological, ecological, and evolutionary components of BD change. Specifically, for individual-based data such as SFC, FD indices should describe changes in the structure of multi-dimensional trait space, where each coordinate axis corresponds to a measured trait and each point represents an individual organism. This representation of BD is analogous to Hutchinsonian niche hypervolume, where species are points in a multi-dimensional space defined by environmental parameters (Hutchinson, 1957). Our proposed approach, therefore, expands the functional niche definition by Rosenfeld (2002), where niche axes are represented by ecological processes that are in fact related to morphological and physiological attributes of individuals (Rosenfeld, 2002).

According to these considerations and those explained in the previous section, we identify FRic, FEve, and three indices of functional divergence (FDiv, FDis, and Rao coefficient) as the most promising existing FD indices for application to individual-based SFC data (Table 1). Their definition (presented earlier) and interpretation can easily be adapted to fit individuals instead of species. It is important to note that trait divergence of individual-based data can potentially be interpreted in the context of evolutionary adaptation: single individuals or groups of similar individuals that diverge from the centroid of the trait distribution can be thought to reflect combinations of traits under selection. Over time such trait divergence processes illustrate evolutionary trajectories that target populations express as a response to an ongoing EC. On the contrary, the widely used species mean traits do not allow to detect differences and changes at the phenotypic level where evolution effectively works, namely in diverging alternative strategies and phenotypes at the individual level.

FRic, FEve, FDiv, FDis, Rao coefficient are based on the objective position of every individual organism (point) within a multi-dimensional trait space. Therefore, these indices do not require a processes of categorization, they allow the use of raw data, and restrict the number of critical decision steps in calculating BD measures (Table 1, Figure 2). As they also do not require calculation of large relative distance matrices among data points or functional dendrograms (Table 1), these indices are directly computable for each sample separately and comparable among samples. These indices and associated functions are freely available for use and calculation using the open source programming language R (R-Development-Core-Team, 2014) through the *FD* package (Laliberté and Legendre, 2010). Our preliminary assessment suggests that they are computationally tractable even for very large high-frequency individual-based SFC datasets (Table 1).

Both the Rao coefficient and FDis seem to embed a mixture of functional divergence and functional trait richness (Mason et al., 2013). From a theoretical point of view, FDiv, FDis, and Rao coefficient could all be suitable for application to individual-based SFC data. Uncertainty however exists on which one would perform best with this type of data sets (Schleuter et al., 2010). We suggest to consider all these divergence measures in a detailed future assessment of their actual performance.

## Concluding remarks and outlook

The main reason for an increasing interest toward a functional description of BD is the expectation that it better describes EF and services than taxonomic diversity (Cardinale et al., 2012). Based on previews empirical and theoretical studies in community ecology, we predict a positive relationship between FD and EF (Ptacnik et al., 2008; Striebel et al., 2009; Cardinale et al., 2012). Very little is known about the exact form of that relationship. New applications of SFC seem to be the most promising way to address those research questions, as we argue that measuring FD at the individual level adds a more realistic understanding of the phenotypic variance in FD, which is the raw material for any filtering or selection process determining the dynamics of response to EC. Therefore, individual level indices should be better applicable for predicting both the future FD and EF. This is especially relevant for predictions of complex cross-generational multi-species community response to changing environmental conditions (e.g., climate change).

We have highlighted some of the limitations of SFC analysis of trait-diversity. We believe that it will be crucial in the future to empirically test how much SFC-derived trait-diversity retain information about the diversity of plankton shapes and strategies, and how they relate to important aquatic ecosystem processes like the production and degradation of organic matter. It will be crucial in the future to test our proposed set of FD indices in simulated artificial and real-world scenarios. A reliable index has to be accurate when replicated both in space and time. It must tractably respond to the changes in the structure of the multivariate trait space as expected by the formal definition of its properties (richness, evenness, and divergence) and from ecological and evolutionary theory (Schleuter et al., 2010). The concepts and conclusions developed here in the context of SFC datasets are general and applicable to individual-level data of any type. Furthermore, ideal indices are applicable to a broad range of groups of organisms, potentially to all organism for which it is possible to measure morphological and physiological continuous (i.e., non-categorical) data at the level of single individuals coexisting in a community.

### Conflict of interest statement

The authors declare that the research was conducted in the absence of any commercial or financial relationships that could be construed as a potential conflict of interest.

## Abbreviations

FD: functional diversity
BD: biodiversity
EC: environmental change
EF: ecosystem functioning
SFC: scanning flow-cytometry
FL: fluorescence.
